# Physical activity for the prevention and treatment of sarcopenic obesity

**DOI:** 10.1002/jcsm.12216

**Published:** 2017-06-20

**Authors:** Masakazu Saitoh, Junichi Ishida, Jochen Springer

**Affiliations:** ^1^ Innovative Clinical Trials, Department of Cardiology and PneumologyUniversity Medical Center GöttingenGöttingenGermany

Sarcopenic obesity was first defined by Baumgartner as the coexistence of sarcopenia and obesity.^1^ A meta‐analysis demonstrated that sarcopenic obesity is associated with a 24% increase in the risk of all‐cause mortality compared to patients without sarcopenic obesity particularly in men.^2^ Moreover, sarcopenic obesity is associated with several adverse outcomes with increased risk of mobility disability, low quality of life, and independence.^3^, ^4^ The multifactorial interactions of common pathophysiological mechanisms underlie the close relationship between sarcopenia and obesity.^5^ Potential mechanisms for the developing of sarcopenic obesity include insulin resistance, increased chronic inflammation, decreased hormones, and decreases in energy expenditure. A recent study has shown that the prevalence of sarcopenic obesity ranged from 1.3 to 11.0% and the range of sarcopenia was 12.6 to 17.5%.^6^ In this study, Tyrovolas *et al*. reported that lower physical activity was significantly associated with sarcopenic obesity, while a dose‐dependent relationship between the number of chronic diseases and sarcopenic obesity was observed.

Physical activity is widely recognized as a means for the primary prevention of chronic disease.^7^, ^8^ Some studies suggested potential health benefits of physical activity and exercise training on sarcopenic obesity.^9^, ^10^ Therefore, breaking this vicious cycle by increasing physical activity could be one of the treatments for prevention and improvement of sarcopenic obesity. In order to make a better understanding of the strategy of patients with sarcopenic obesity, evaluation of the physical activity is no less important than assessment of muscle mass, muscle strength, and physical performance. It remains unclear whether increasing physical activity affects sarcopenic obesity; much more evidence of longitudinal studies will be needed until we can confirm if physical activity would be an important therapeutic target in patients with sarcopenic obesity.
